# Secondary Atomization and Micro-Explosion Effect Induced by Surfactant and Nanoparticles on Enhancing the Combustion Performance of Al/JP-10/OA Nanofluid Fuel

**DOI:** 10.3390/molecules29081806

**Published:** 2024-04-16

**Authors:** Shengji Li, Zixuan Liu, Qianmei Yang, Zhangtao Wang, Xuefeng Huang, Dan Luo

**Affiliations:** 1 College of Materials and Environmental Engineering, Hangzhou Dianzi University, Hangzhou 310018, China; shengjili@hdu.edu.cn (S.L.);; 2 Institute of Energy, Department of Physics, Hangzhou Dianzi University, Hangzhou 310018, China

**Keywords:** nanofluid fuels, aviation fuels, combustion, micro-explosion, concentration effect

## Abstract

Aluminum/tetrahydrodicyclopentadiene/oleic acid (Al/JP-10/OA) nanofluid fuel is considered a potential fuel for aircraft powered by aviation turbine engines. However, an optimized formula for an Al/JP-10/OA system inducing a secondary atomization and micro-explosion effect and improving the burning performance needs to be developed. With this aim, in this work, the combustion characteristics of pure JP-10, JP-10/OA, JP-10/Al, and Al/JP-10/OA were experimentally tested, and a comparative analysis was conducted. Specifically, the influence of the surfactant and nanoparticle concentrations on the combustion characteristics of Al/JP-10/OA nanofluid fuel, including the flame structure, the flame temperature, the burning rate, the secondary atomization and micro-explosion effect, etc., were evaluated in detail. The results demonstrate that the addition of OA surfactant and Al nanoparticles had a significant effect on the burning rate of fuel droplets. The OA had an inhibition effect, while the Al nanoparticles had a promotion effect. As both OA and Al nanoparticles were added to the JP-10, the synergetic effect had to be considered. At the optimum ratio of OA to Al for the best suspension stability, there is a critical Al concentration of 1.0 wt.% from promotion to inhibition with increases in the Al concentration. The addition of OA and Al nanoparticles induced the secondary atomization and micro-explosion, resulting in an unsteady combustion and chaotic flame structure. The transient flame temperature of hundreds of Kelvins increased, the high-temperature flame zone widened, and thus, the energy release was elevated. Therefore, the combustion performance and energy release of Al/JP-10/OA nanofluid fuel can be improved through the secondary atomization and micro-explosion effect induced by the surfactant and nanoparticles.

## 1. Introduction

In the aviation industry, with the increasing depletion of non-renewable aviation fuels and the emissions of a large number of pollutants, great attention is being paid to the utilization of low-CO_2_-emission fuels [1] and improving the utilization efficiency of aviation fuels [2,3]. Simultaneously, the problem of how to improve the fuel performance and increase the flight range of aircraft using equal amounts of fuel, due to the limited storage volume of the fuel systems of aircraft, will have to be faced [4,5,6]. To solve this problem, one of best strategies is to add highly energetic solid nanoparticles to the liquid fuels to form nanofluid fuels [7]. Nanofluid fuels have many advantages, such as high energy density, high thermal conductivity, high ignition probability, high combustion efficiency, and low pollutant emissions compared to the base fuels [8,9,10].

Gao et al. [11] reported the combustion characteristics of nanofluid fuel droplets prepared from polydopamine (PDA)-coated nano-aluminum and copper (II) oxide composites and kerosene and found that the kerosene droplets with nano-Al particles had the shortest ignition delay time, and the nano-Al/PDA/CuO (2%) kerosene droplets had the highest combustion rate. Venu et al. [12] prepared Al_2_O_3_-PBD biodiesel with concentrations of 25 ppm and 50 ppm and tested its combustion and emission characteristics. It was found that the addition of nanoparticles improved the combustion efficiency of the base fuel and reduced the pollutant emissions. Yu et al. [13] demonstrated that adding Al nanoparticles to the JP-10 base fuel enhanced the combustion efficiency of the fuel by 3.0~9.0%. Javed et al. [14] testified that adding a low concentration of Al nanoparticles to kerosene could reduce the ignition delay and temperature of the kerosene droplets and increase the combustion rate of the droplets, leading to the occurrence of micro-explosion and reducing the burn time of the kerosene droplets.

Among aviation fuels, JP-10 (C_10_H_16_, exotetrahydrodicyclopentadiene) has been widely used in aircraft with aero turbine engines [15,16], with a volumetric heat of 39.6 MJ/L, a freezing point of <−100 °C, and kinematic viscosity of 19 cSt at −40 °C [17,18]. Energetic Al nanoparticles are a type of metal fuel that has been applied in propellants as an additive due to its characteristics of a higher energy density and a low price [19,20]. Correspondingly, to enhance the dispersion stability of Al nanoparticles in JP-10, a surfactant is required. The surfactant with the best effect on the stability enhancement of the Al/JP-10 system is OA [21]. Therefore, in this work, the Al/JP-10/OA nanofluid fuel system was selected to conduct experiments and further evaluate its combustion characteristics.

For the Al/JP-10/OA nanofluid fuel system, one conflict is that there are different Al concentrations for obtaining the best suspension stability, physical properties, evaporation, and micro-explosion characteristics, as demonstrated in our previous work [21]. Similarly, the concentrations of OA surfactant and Al nanoparticles possibly play an important role in influencing the combustion performance. Therefore, the optimum concentrations of OA surfactant and Al nanoparticles are crucial to balance these characteristics. Another contradiction of the B/JP-10/SP-80 nanofluid fuel system was found in the reduction of the burning rate in the stable combustion stage and the shortening of its lifetime due to the repeated occurrence of secondary atomization and micro-explosion in the late combustion stage, as shown in another work [22]. Obviously, the secondary atomization and micro-explosion make the burning mother droplets break up into finer daughter droplets, thus increasing the surface area-to-volume ratio and bringing nanoparticles into the gas-phase flame zone, which are helpful for enhancing the energy release of fuels and promoting the heat transfer, including the thermal conduction and radiation feedback. This motivates us to carry out experimental works on the secondary atomization and micro-explosion effect induced by the surfactant and nanoparticles on enhancing the combustion performance of Al/JP-10/OA nanofluid fuel.

In this work, the emphasis was on the comparative analysis of burning base fuels JP-10, JP-10/OA, JP-10/Al, and Al/JP-10/OA, and the differences in their combustion process, flame structure, flame temperature, burning rate, burn time, secondary atomization and micro-explosion intensity, temperature variation before and after secondary atomization and micro-explosion were evaluated in detail. The best formula for the Al/JP-10/OA nanofluid fuel system to improve its combustion performance was correspondingly obtained, laying the foundation for subsequent combustion performance improvement in practical aircraft engines.

## 2. Results and Discussion

### 2.1. Combustion Evolution of Pure JP-10 Droplets

**Figure 1:**
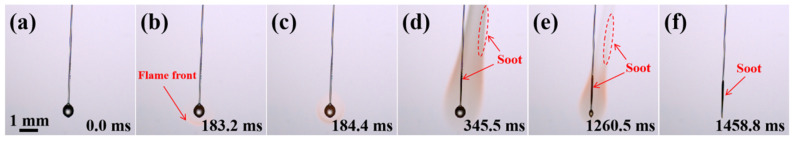
The combustion evolution of a pure JP-10 droplet. (**a**) 0.0 ms, (**b**) 183.2 ms, (**c**) 184.4 ms, (**d**) 345.5 ms, (**e**) 1260.5 ms, (**f**) 1458.8 ms.

Figure 1 shows the burning process of a pure JP-10 droplet with the aid of backlight illumination. It can be clearly observed that in high-temperature air, the JP-10 droplet was ignited after being heated for about 183.2 ms (Figure 1b). The ignition first occurred at the bottom of the droplet near the nickel chromium wire coil, according to the flame shaped like a crescent (Figure 1b). Then, the flame propagated along the droplet surface and surrounded the droplet (Figure 1c). The flame structure transformed from the crescent into a teardrop, but their flame structures were symmetrical. The diffusion-controlled flame was transparent, and the flame thickness was relatively thin, suggesting that the combustion was complete. With the evolution of combustion, the flame structure became slimmer, and the flame thickness increased. Simultaneously, it is worth noting that the soot formed within the flame zone (Figure 1d). JP-10, a hydrocarbon fuel, can incompletely burn during combustion due to an insufficient oxygen supply around the droplet, resulting in soot formation [23]. The soot partially dispersed in the air, and partially attached to the quartz fiber (Figure 1e,f). After that, the pure JP-10 fuel droplets maintained quite stable combustion until the JP-10 fuel completely burned (see Video S1 in the Supporting Information (SI) for high-speed images and corresponding temperature variations).

### 2.2. Combustion Evolution of JP-10/OA Droplets

In order to ensure the long-term suspension stability of nanofluid fuels in practical engineering applications, it is necessary to add a certain amount of surfactant into the base liquid to disperse the nanoparticles during preparation. In this work, the OA surfactant was added to JP-10 to disperse the Al nanoparticles. This is no doubt that OA affects the combustion characteristics of nanofluid fuels. Therefore, experiments on different OA concentrations (0.2~12.5 wt.%) influencing the combustion characteristics were first conducted. The various OA concentrations just met the requirements for the best suspension stability of the Al concentration (0.1~5.0 wt.%). 

**Figure 2:**
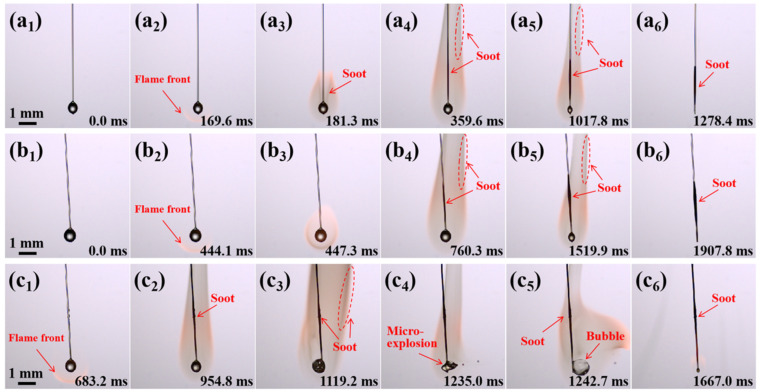
The combustion evolution of JP-10+OA0.2 (**a_1_**–**a_6_**), JP-10+OA2.0 (**b_1_**–**b_6_**), and JP-10+OA12.5 (**c_1_**–**c_6_**) droplets.

Figure 2 shows the combustion evolution of a JP-10/OA droplet prepared by adding 0.2 wt.%, 2.0 wt.%, 12.5 wt.% OA to the JP-10 (see Videos S2–S4 in the SI for image and temperature data) as examples. For the JP-10+OA0.2 droplets (Figure 2(a_1_–a_6_)), it was found that the ignition delay time (169.6 ms) was slightly shortened, but the burning process was the same as that of the JP-10 droplet. For the JP-10+OA2.0 (Figure 2(b_1_–b_6_)) and JP-10+OA12.5 (Figure 2(c_1_–c_6_)) droplets, their ignition delay time (444.1 ms, 683.2 ms) was obviously enlarged, and the burn time became longer, compared to the JP-10. This suggests that the addition of OA surfactant with higher concentrations inhibited the heat absorption from the high-temperature air because of its lower thermal conductivity, resulting in a reduction in the evaporation and burning rate (see Section 2.5). 

It can be observed that the combustion phenomenon of the JP-10+OA12.5 droplets at the early stage was the same as the JP-10, JP-10+OA0.2, and JP-10+OA2.0 droplets. The flame structure and profile were similar, demonstrating the symmetrical characteristic. As the combustion proceeded, it is worth noting that bubble formation, breakup, and secondary atomization and micro-explosion were observed, as shown in Figure 2(c_3_–c_5_). Moreover, the secondary atomization and micro-explosion process occurred repetitively. This means that as the OA concentration increased, the combustion evolution of the JP-10/OA droplets presented different phenomena, i.e., secondary atomization (weak level, sprayed toward one direction), even micro-explosion (strong level, sprayed toward multiple directions). There is no doubt that the secondary atomization and micro-explosion made the mother droplets break up into finer daughter droplets, and thus reduced the size and shortened the lifetime of the mother droplets. However, this does not mean that the lifetime of the JP-10+OA12.5 droplets is shorter than that of the JP-10 droplets with the same size. Although the secondary atomization and micro-explosion of the JP-10+OA12.5 droplets increased the specific surface area for evaporation and burning, the burning rate and the lifetime suffered from the influence of other factors, like the boiling point, thermal conductivity, etc.

Generally, the occurrence of secondary atomization and micro-explosion is due to the difference in the boiling points of JP-10 and OA. As the combustion evolves, the OA, with a higher boiling point, will wrap around the surface of the droplet to form an OA molecular layer. If the temperature inside the droplet is higher than the boiling point of JP-10, the JP-10 will vaporize, uniformly nucleate and form bubbles inside the droplet. The heat absorbed from the high-temperature air makes the internal pressure of the bubbles increase and the droplet expand. When the internal pressure of the bubbles exceeds the surface tension of the droplet, the droplet breaks up, possibly causing secondary atomization or micro-explosion. This phenomenon was also observed during the combustion process of droplets with sorbitol anhydride oleate as a surfactant and n-decane as the base liquid [24].

The comparison among the JP-10+OA0.2, JP-10+OA2.0, and JP-10+OA12.5 droplets demonstrates that the OA concentration plays a crucial role in the occurrence of secondary atomization or micro-explosion. The results show that secondary atomization or micro-explosion can be observed when the OA concentration is equal to or more than 5.0 wt.% (see Section 2.7), while this seldom occurs when the OA concentration is below 5.0 wt.%.

### 2.3. Combustion Evolution of JP-10/Al Droplets

**Figure 3:**
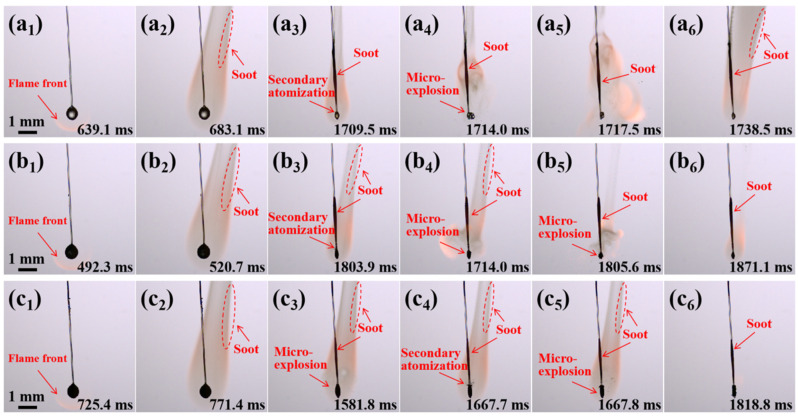
The combustion evolution of JP-10+Al0.1 (**a_1_**–**a_6_**), JP-10+Al1.0 (**b_1_**–**b_6_**), and JP-10+Al5.0 (**c_1_**–**c_6_**) droplets.

To identify the sole contribution of the Al nanoparticles to the micro-explosion, the combustion evolution of JP-10/Al droplets was experimentally observed, as shown in Figure 3 (see Videos S5–S7 in the SI for image and temperature data). Despite low Al concentrations of 0.1 wt.% and 1.0 wt.% or high Al concentrations of 5.0 wt.%, after the droplets experienced a stable combustion, bubbles formed and gradually expanded inside the JP-10/Al droplets in the late stage, leading to the occurrence of intensive secondary atomization and micro-explosion (Figure 3(a_3_–a_6_,b_3_–b_6_,c_3_–c_6_)). The flame structure and profile dramatically oscillated. However, the micro-explosion intensity of the JP-10+Al5.0 droplets was larger than those of the JP-10+Al0.1 and JP-10+Al1.0 droplets, which is discussed in Section 2.7. This suggests that the Al concentration influences the micro-explosion behavior, since the content of Al nanoparticles dispersed throughout the JP-10 comprehensively affected the heat absorption from the high-temperature air and the formation of an Al shell at the droplet surface.

It is worth noting that the moment of occurrence of secondary atomization and micro-explosion for the JP-10/Al droplets was obviously later than that of JP-10/OA, and bubbles were seldom observed. Since JP-10/OA is a liquid–liquid two-phase system, its secondary atomization and micro-explosion are induced by the difference in the boiling points between the two; meanwhile, JP-10/Al is a liquid–solid two-phase system, and its secondary atomization and micro-explosion occur at the drying stage. This means that the Al concentration is so high that the Al nanoparticle aggregates form an inhibition shell inside the droplet, and the gas-phase JP-10 builds up pressure inside the shell. When the pressure exceeds the droplet surface tension and shell stress, it will result in the occurrence of secondary atomization and micro-explosion. 

### 2.4. Combustion Evolution of Al/JP-10/OA Nanofluid Fuel Droplets

**Figure 4:**
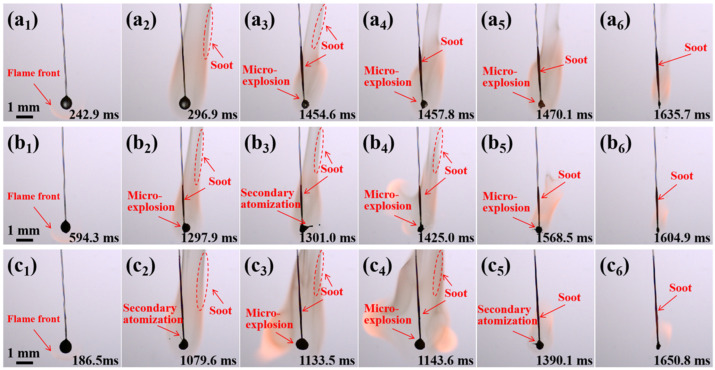
The combustion evolution of JP-10+OA+Al0.1 (**a_1_**–**a_6_**), JP-10+OA+Al1.0 (**b_1_**–**b_6_**), and JP-10+OA+Al5.0 (**c_1_**–**c_6_**) droplets.

Figure 4 shows the combustion phenomenon of the Al/JP-10/OA nanofluid fuel droplets with different Al concentrations (see Videos S8–S10 in the SI for image and temperature data). The whole combustion process can be divided into four stages, i.e., preheating and ignition; stable combustion of JP-10 and OA surfactant, followed by d^2^ law; oscillating combustion, including secondary atomization and micro-explosion; and metallic Al combustion. This is different from the combustion process reported by Gan et al. [24]. They found that the combustion of Al/n-decane/SP-80 nanofluid fuel droplets included five distinctive stages: preheating and ignition, classical combustion, micro-explosion, surfactant flame, and aluminum droplet flame. The comparison between the two nanofluid fuel systems suggests that the surfactants with different boiling points burn at different stages.

It can be clearly seen that the evolution of the burning Al/JP-10/OA nanofluid fuel droplets experienced secondary atomization and micro-explosion at each Al concentration. The Al/JP-10/OA nanofluid fuel droplets with low concentrations of Al nanoparticles (0.1 wt.%) demonstrated weak micro-explosions (Figure 4(a_1_–a_6_)); however, at higher Al concentrations, the micro-explosions became more intensive (Figure 4(b_1_–b_6_,c_1_–c_6_). Compared to JP-10/OA and JP-10/Al, the secondary atomization and micro-explosion of Al/JP-10/OA occurred in advance. Al/JP-10/OA is a liquid–liquid–solid multiphase system; the OA surfactant and Al nanoparticles have a synergetic effect on the whole process of heat absorption, nucleation, bubble formation and expansion, shell formation and compactness, and secondary atomization and micro-explosion. 

### 2.5. Combustion Rate Analysis

For burning JP-10, JP-10/OA, JP-10/Al, and Al/JP-10/OA fuel droplets, their combustion rates can be obtained by measuring the variation in the droplet square diameter with the evolution time during the whole combustion stage. If the droplets undergo secondary atomization and micro-explosion, the effective burning rate can be fitted during the stable combustion according to the classical *d*^2^-law of droplet combustion [22]:

(1) $$\frac{d^{2}}{d_{0}^{2}}=-K_{b}\frac{t}{d_{0}^{2}}+1$$

in which *d* and *d*_0_ are the instantaneous and initial droplet diameters, respectively. *K_b_* is the burning rate.

**Figure 5:**
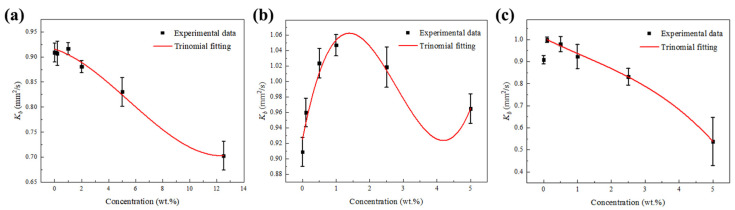
The variation in the effective burning rate of pure JP-10, JP-10/OA (**a**), JP-10/Al (**b**), and Al/JP-10/OA (**c**) fuel droplets with the corresponding concentrations.

The evolving normalized square diameter of the pure JP-10 and JP-10/OA droplets with different concentrations of surfactant varied with the normalized time. It was found that the normalized square diameter of the JP-10/OA droplets with low concentrations of OA were linearly reduced over time, which is consistent with the change in the pure JP-10 droplets and follows the classical *d*^2^ law. However, the JP-10/OA droplets with high concentrations of OA experienced expansion and secondary atomization and micro-explosion during combustion, and thus, the droplet size change fluctuated. After each micro-explosion, the size of the mother droplet became smaller than before. As the OA concentration increased, the burning rate of the droplet in the stable combustion stage decreased, and the intensity of droplet micro-explosion became more severe. Figure 5a shows the burning rate of the stable combustion stage of the JP-10/OA droplets. It can be seen that the combustion rate of the JP-10/OA droplets with an OA concentration below 2.0 wt.% was almost equivalent to that of pure JP-10. When the concentration of the OA surfactant exceeded 2.0 wt.%, the combustion rate of the JP-10/OA droplets significantly decreased with the increase in the surfactant concentration. This proves that the addition of a low concentration of OA had little effect on the combustion rate of the droplets, which is consistent with the results obtained by Javed [25]. They found that the combustion rate of kerosene droplets with 0.25 wt.% and 0.5 wt.% OA addition at 700–800 °C was almost the same as that of pure kerosene. However, in this work, it was found that adding OA surfactant at high concentrations resulted in a large reduction in the combustion rate of the droplets, 8.7% @ 5.0 wt.% OA, 22.7% @ 12.5 wt.% OA. This is because the boiling point of JP-10 is much lower than that of OA. During the combustion process, the OA wraps around the surface of the droplets to form a molecular film. The molecular film largely inhibits the evaporation of JP-10. The higher the concentration of OA, the thicker the molecular layer formed on the surface of the droplets, and thus, the greater the reduction in the droplet combustion rate [26].

Figure 5b shows the variation in the combustion rate of JP-10/Al droplets with the Al concentration. The combustion rates of the JP-10/Al droplets were much higher than that of pure JP-10, indicating that the addition of Al nanoparticles had a promoting effect on the combustion rate of the JP-10. Especially for the JP-10/Al droplets with a 1.0 wt.% Al concentration, the combustion rates of the droplets enhanced by 15.2%, compared to the pure JP-10 droplets. It is worth noting that the Al concentration did not monotonically influence the combustion rate. According to the trinomial fitting between the combustion rate and the Al concentration ranging from 0~5.0 wt.%, there is a maximum combustion rate at an Al concentration of ~1.5 wt.%. When the concentration of Al nanoparticles is below 1.5 wt.%, the combustion rate of the droplets increases with increases in the Al nanoparticle concentration. This is because adding Al nanoparticles can enhance the radiation and heat transfer of the droplets [25,27], thereby enhancing the combustion rate of the droplets. When the concentration of Al nanoparticles is beyond 1.5 wt.%, the combustion rate of the droplet decreases with an increasing Al nanoparticle concentration. This is possibly due to the agglomeration and aggregation of Al nanoparticles inside the droplets without the aid of a surfactant [21]. This trend is consistent with the influence of the nanoparticle concentration on the combustion rate of nano-graphene/refined kerosene nanofluid fuel without surfactant reported by Yadav et al. [28]. They found that the combustion rate of refined kerosene droplets increased most significantly when the concentration of nano-graphene was 0.2 wt.%. When the concentration of nanoparticles exceeded this value, nano-graphene flakes were agglomerated, and the combustion rate of droplets was reduced.

For Al/JP-10/OA nanofluid fuel droplets with different Al concentrations, the normalized square diameters at each concentration significantly oscillated due to secondary atomization or micro-explosion. It was astonishingly found that the effective burning rate of Al/JP-10/OA droplets during the stable combustion stage generally monotonically decreased with increases in the Al concentration, as shown in Figure 5c. Compared to pure JP-10, the combustion rate of Al/JP-10/OA nanofluid fuel droplets with a 0.1 wt.% Al concentration increased by 10.0%. When the concentration of Al nanoparticles ranged from 0.1 wt.% and 1.0 wt.%, the combustion rate of the Al/JP-10/OA nanofluid fuel droplets decreased with the increasing Al concentration, but was always higher than the combustion rate of the JP-10 droplets. When the Al concentration was greater than 1.0 wt.%, the combustion rate of the Al/JP-10/OA nanofluid fuel droplets decreased with the increase in the Al concentration but was lower than the combustion rate of the JP-10 droplets (8.5% reduction @ 5.0 wt.% OA+2.5 wt.% Al, 40.8% reduction @ 12.5 wt.% OA+5.0 wt.% Al). Therefore, at the optimum ratio of OA to Al for the best suspension stability, there is a critical Al concentration for the adjustment of the combustion rate of Al/JP-10/OA nanofluid fuel. By comparing the differences in the combustion rates among JP-10, JP-10/OA, JP-10/Al, and Al/JP-10/OA, it can be testified that the addition of the OA surfactant and Al nanoparticles had a synergistic effect on the modulation of the combustion rate. Although the addition of OA surfactant reduced the combustion rate of JP-10, the addition of Al nanoparticles was beneficial for increasing the combustion rate, resulting in a combined effect. This phenomenon was also found by Javed et al. [26] when investigating the combustion characteristics of an Al/kerosene nanofluid fuel system. They found that the combustion rate of liquid droplets at the ambient temperature of 600~800 °C first increased and then decreased at 0.1 wt.%~1.0 wt.% concentrations of added Al nanoparticles. The corresponding Al concentration to reach the maximum combustion rate of the Al/kerosene nanofluid fuel was at 0.5 wt.%, and the ratio of Al to OA was 2:1, while in this work, the concentration was 1.0 wt.%, and the ratio of Al to OA was 1:2 for the Al/JP-10/OA nanofluid fuel. At the same Al concentration, if the content of OA increased, a stronger inhibitory effect will occur on the droplets’ combustion, leading to a reduction in the combustion rate.

Simultaneously, it is noted that our previous work [21] deeply revealed the contradiction of the concentration effect on the stability, physical properties, evaporation, and micro-explosion characteristics and obtained the best Al and OA concentrations to maintain the most suitable comprehensive performance. Therefore, the best formula for a Al/JP-10/OA nanofluid fuel system to improve the combustion performance must comprehensively consider and balance multiple aspects in practical engineering applications. 

### 2.6. Flame Temperature Analysis

**Figure 6:**
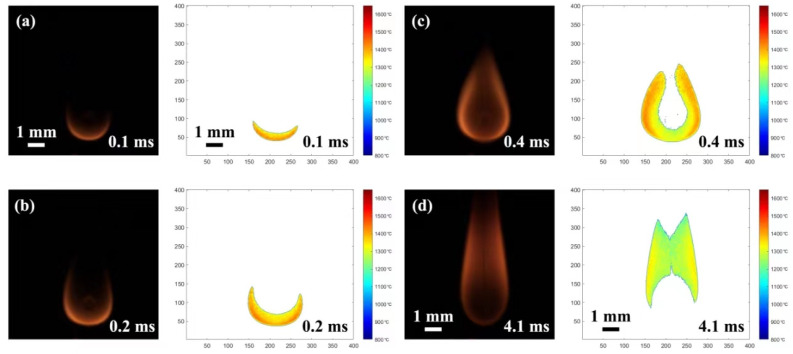
Temperature distribution of combustion flame of pure JP-10. (**a**) 0.1 ms, (**b**) 0.2 ms, (**c**) 0.4 ms, (**d**) 4.1 ms.

**Figure 7:**
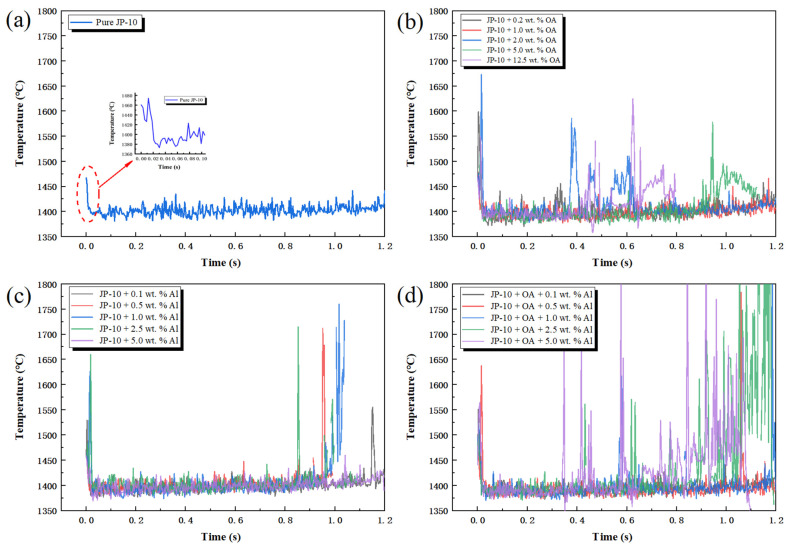
Temperature histories of burning JP-10 (**a**), JP-10/OA (**b**), JP-10/Al (**c**), and Al/JP-10/OA (**d**) droplets.

Figure 6 shows the flame temperature distribution of the JP-10 droplet during combustion. The high-speed images and corresponding temperature variation can be found in Video S1 in the SI. Firstly, it can be observed that the temperature of the droplet bottom was higher that of the droplet top, meaning that ignition occurred at the high-temperature zone near the heating coil. Then, the whole droplet was gradually surrounded by the flame, and the temperature near the flame front was obviously higher than that of the inner flame. The highest flame temperature of the burning droplet was ~1475 °C during the whole combustion process, which occurred near the flame front profile at the ignition moment (Figure 7a). After ignition, the highest temperature during the stable combustion stage was above 1380 °C and below 1420 °C (Figure 7a). This is in agreement with the results demonstrated by Yadav et al. [28], who measured the highest temperature of the kerosene droplet combustion flame using an infrared camera at ~1400 °C. It also verifies that the temperature measurement based on the two-color radiation methodology by analyzing the images acquired by a high-speed camera is acceptable. It is worth noting that there was a peak temperature (1475 °C) at the ignition moment, which was higher by ~70 °C than the stable combustion temperature. This is because the oxidation reaction led to a large amount of heat release, elevating the system’s temperature, and then the heat loss to the surrounding air after ignition induced the temperature decrease in the system. 

The variation in the highest flame temperature of the JP-10, JP-10/OA, and JP-10/Al, Al/JP-10/OA droplets was extracted to analyze the differences among the temperature evolution of combustion, as shown in Figure 7. The results indicate that the highest flame temperature during the ignition stage of droplets was higher than that during the stable combustion stage. The flame temperature nearby the droplet surface was the lowest, which is consistent with the results obtained by Gao et al. [11] and Ao et al. [29]. They believe that precursors and aggregates of soot are easily formed on the surface of the droplet and the edge of the flame, while the concentration of soot at the top of the flame is higher, resulting in lower flame temperatures in these areas. The combustion stage of Al agglomerates included in the combustion process of the JP-10/Al and Al/JP-10/OA droplets was not measured due to overexposure.

### 2.7. Secondary Atomization and Micro-Explosion Analysis

#### 2.7.1. Evolution of Secondary Atomization and Micro-Explosion

Secondary atomization and micro-explosion of fuel droplets can break up the mother droplets into a large number of finer daughter droplets and distribute them in a high-temperature flame zone, which makes the base fuel, the surfactant, and nanoparticles burn more completely and improves the combustion performance. Generally, the secondary atomization and micro-explosion of burning fuel droplets depend on the internal pressure inside the droplets, which relates to the completeness and compactness of the shell formed by aggregating the surfactant and nanoparticles [22,30,31]. 

For JP-10/OA droplets at low OA concentrations (below 2.0 wt.%), during combustion, the OA layer on the surface of the droplets could not inhibit the evaporation of JP-10, and thus, secondary atomization was not observed. When the OA concentration increased, the thickness of the OA layer on the surface enlarged, leading to the enhancement of the inhibition effect and the occurrence of secondary atomization, and even micro-explosion (Figure 2). For JP-10/Al droplets, secondary atomization and micro-explosion occurred at each Al concentration between 0.1 wt.% and 5.0 wt.% (Figure 3). This is because, at low concentrations, Al nanoparticles tend to move to the inner surface of droplet and produce a barrier layer to build pressure inside the droplet, resulting in secondary atomization and micro-explosion. Meanwhile, at higher concentrations, the aggregate of Al nanoparticles cannot form a perfect barrier layer, and the inhibition of the nano-Al layer is weakened. For the Al/JP-10/OA droplets, due to the synergetic effect of the surfactant and nanoparticles, the barrier layer on the droplet surface became thicker and denser, and secondary atomization and micro-explosion at all concentrations between 0.1 wt.% and 5.0 wt.% occurred (Figure 4).

#### 2.7.2. Intensity of Secondary Atomization and Micro-Explosion

The formation of the barrier layer within the droplet significantly affects its diffusion and evaporation, which are dependent of the movement of the surfactant and nanoparticles inside the droplets, the formation of nucleation sites, the transport of particles to the droplet surface, and the aggregation of the particles. The barrier layer is helpful for building pressure inside the droplet, leading to secondary atomization and micro-explosion. 

**Figure 8:**
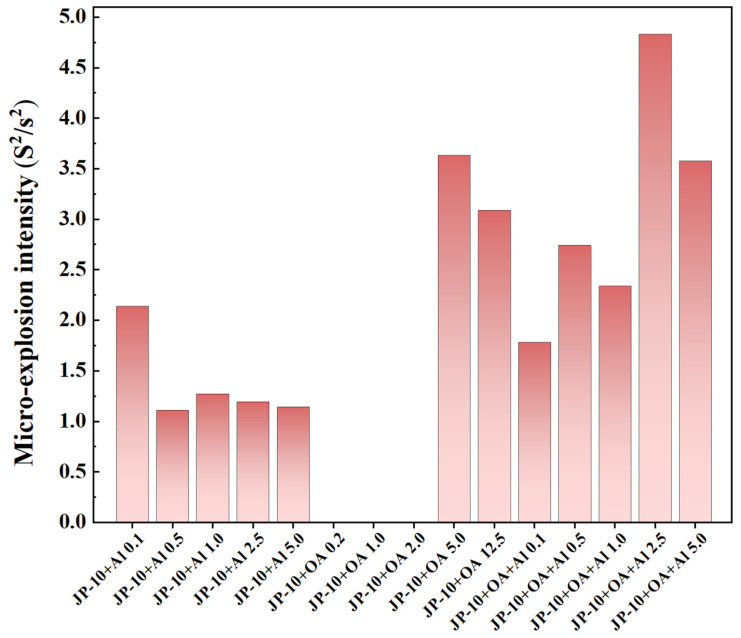
Intensity of secondary atomization and micro-explosion of burning JP-10/OA, JP-10/Al, and Al/JP-10/OA droplets.

Considering the uncertainty and randomness of the micro-explosion of burning droplets, the secondary atomization and micro-explosion intensity (I) should be defined as the mass ratio of the mother droplet before and after the secondary atomization and micro-explosion. Meanwhile, for the two-dimensional images acquired by the high-speed camera, using the imaging treatment technique, the intensity can be only calculated by the ratio of the projected mother droplet area before and after the secondary atomization and micro-explosion to quantitatively evaluate the phenomenon. Figure 8 demonstrates the maximum intensity of secondary atomization and micro-explosion of burning JP-10/OA, JP-10/Al, and Al/JP-10/OA droplets. It was found that the fuel droplets containing Al nanoparticles produced secondary atomization and micro-explosion, while the fuel droplets containing OA at concentrations below 2.0 wt.% did not. It should be noted that the intensity of secondary atomization and micro-explosion of the JP-10/Al droplets was weak, smaller than 2.0. The intensity of secondary atomization and micro-explosion of the Al/JP-10/OA droplets was relatively high; most of them were higher than 2.0. This comparison suggests that the addition of OA is helpful for improving the intensity of secondary atomization and micro-explosion, simultaneously guaranteeing the suspension stability.

#### 2.7.3. Temperature Variation before and after Secondary Atomization and Micro-Explosion

**Figure 9:**
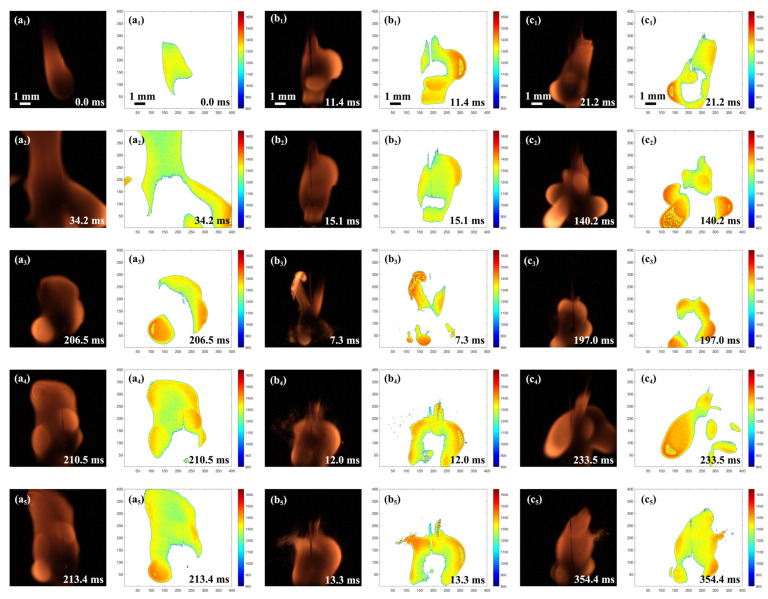
Temperature distribution of secondary atomization and micro-explosion of burning JP-10/OA, JP-10/Al, and Al/JP-10/OA droplets. (**a_1_**–**a_5_**) JP-10+OA12.5, (**b_1_**,**b_2_**) JP-10+Al0.1, (**b_3_**–**b_5_**) JP-10+Al1.0, (**c_1_**) JP-10+OA+Al0.1, (**c_2_**,**c_3_**) JP-10+OA+Al1.0, and (**c_4_**,**c_5_**) JP-10+OA+Al5.0.

Figure 9 shows the temperature distribution before and after secondary atomization and micro-explosion of burning JP-10/OA, JP-10/Al, and Al/JP-10/OA droplets. Obviously, the temperature increased, and the high-temperature zone became wider. This suggests that secondary atomization and micro-explosion changed the stable combustion status. Daughter droplets were ejected from the mother droplets, resulting in an increase in the specific surface area of droplet evaporation, and the expansion of the mixed zone between the fuel vapor and air. More nanoparticles entered into the flame zone, guaranteeing quicker and higher energy release [32].

**Figure 10:**
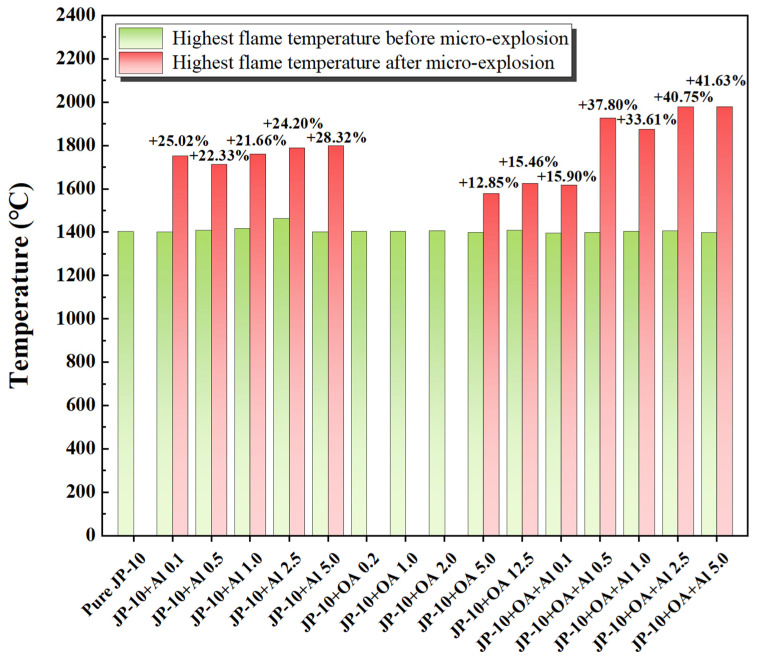
Temperature variation in secondary atomization and micro-explosion of burning JP-10/OA, JP-10/Al, and Al/JP-10/OA droplets.

The temperature variation before and after secondary atomization and micro-explosion is shown in Figure 10. It can be seen that, compared to the stable flame temperature (~1400 °C), the highest temperature in the secondary atomization and micro-explosion zones increased by 12.85~41.36%. With the increase in the Al nanoparticle concentration, the flame temperature can be greatly increased. Specifically, for Al/JP-10/OA nanofluid fuels with an Al nanoparticle concentration of 0.5 wt.% and above, the temperature enhancement can reach over 30%.

## 3. Materials and Experimental Methods

### 3.1. Preparation of Al/JP-10/OA Nanofluid Fuels

The materials used in this work and the preparation method for the nanofluid fuel are consistent with those in our previous work [21]. Al nanoparticles with a median size of 50 nm were selected as the additive, OA was used as the surfactant, and JP-10 was used as the base fuel. The preparation of JP-10/OA and JP-10/Al was based on the one-step method. The weighed OA and Al were directly added to the measured JP-10 and stirred and sonicated at a constant temperature (155 °C) for 20 min to maintain excellent dispersion. 

For the preparation of the Al/JP-10/OA nanofluid fuel, a certain amount of Al nanoparticles was mixed with OA and sonicated for 1 h. Then a certain amount of JP-10 base fuel was added to the mixture and sonicated for 20 min. During sonication, an ice bath was used to keep the nanofluid fuels at 155 °C. In order to maintain the best dispersion stability of Al/JP-10/OA nanofluid fuels, the best mass ratio of Al to OA is 1:2 for 0.1 wt.%, 0.5 wt.%, 1.0 wt.%, and 2.5 wt.% Al concentrations, while the best mass ratio of Al to OA is 1:2.5 for nanofluid fuels with a 5.0 wt.% Al concentration and above. These prepared fuels were named to distinguish the differences between the OA surfactant and Al nanoparticle concentrations, as listed in Table 1.

### 3.2. Experimental Setup for Combustion Characterization

**Figure 11:**
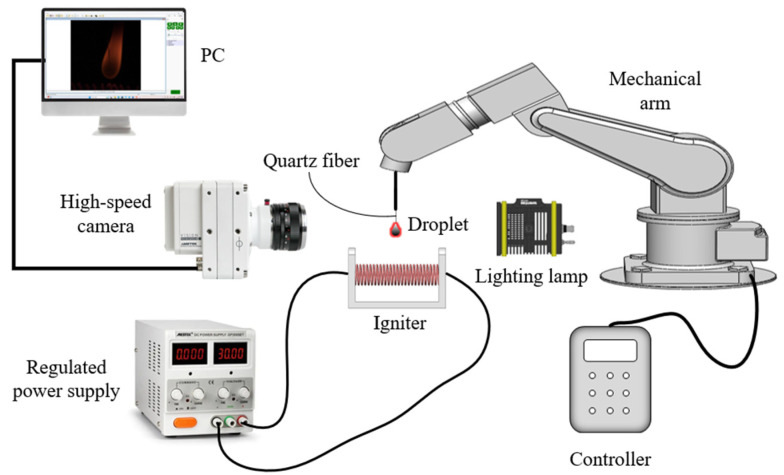
Schematic of experimental setup.

Figure 11 shows a schematic of the experimental setup to characterize the combustion characteristics of the individual nanofluid fuel droplets, including a suspended droplet module, ignition module, illumination module, high-speed imaging, and a temperature measurement module. Herein, a simple description is introduced; the complete introduction can be found in our previous work [22]. Fuel droplets were first generated by extruding the fuel with a needle with an outer diameter of 0.5 mm connected to a micro-syringe of 1.0 mL, and then they were transferred to a quartz fiber with a tiny diameter of 125 μm and low thermal conductivity of 1.4 W/m·K. The droplets were finally suspended at the end of the fiber. The quartz fiber was installed on a mechanical arm with an accuracy of 50 μm. By manipulating the mechanical arm, the droplets could be positioned in the desired high-temperature air zone. The suspended droplets looked elliptic due to the action of gravity and surface tension, and their equivalent diameters stayed at 1.1 ± 0.1 mm. Thus, the influence of the droplet size and morphology on the combustion characteristics could be minimized.

The high-temperature air zone was formed by electrically heating the nickel chromium wire coil. The power of the nickel chromium wire coil could be controlled by an externally regulated power supply. The maximum temperature in the high-temperature air zone was 800 °C, while there was a temperature gradient along the axial direction perpendicular to the nickel chromium wire coil. Therefore, to maintain the same ignition zone, the droplets had to be positioned by controlling the mechanical arm to complete the automatic position after setting the same end point of the quartz fiber.

Backlight illumination, located on the same axis as the high-speed camera, was provided to produce a uniform bright view field to help acquire the images of droplets and their flames using a high-speed camera (Phantom Micro M310). A lens was installed in front of the high-speed camera to magnify the images. During the experiments, the high-speed camera was set to record the evolving flames of the burning droplets at a frame ratio of 10,000 fps with a resolution of 512 × 512 pixels. The sizes of droplets and flame profiles were measured by the digit image processing technique, including cropping, filtering, binarization, contour extraction, calibration, and perimeter and area measurement. Finally, for the droplets that looked ellipsoidal or non-spherical due to deformation, a characteristic equivalent diameter d was defined and calculated by *d* = 4*S_d_/L_d_*, where *S_d_* and *L_d_* represent the area and the perimeter of droplet, respectively. 

In this work, the measurement of the flame temperature was based on the two-color radiation methodology by analyzing the images acquired by the high-speed camera, whose principle and validation procedure is included as follows. 

The response in the central wavelengths of the red, green, and blue channels of the high-speed camera at each pixel point can be expressed as:

(2) $$\left\{\begin{array}{l} R=4\times 10^{-7}\cdot A\cdot K_{T}(\lambda_{r})Y(\lambda_{r})L(\lambda_{r},T)=K_{T}(\lambda_{r})K_{r}L(\lambda_{r},T)\\ G=4\times 10^{-7}\cdot A\cdot K_{T}(\lambda_{g})Y(\lambda_{g})L(\lambda_{g},T)=K_{T}(\lambda_{g})K_{g}L(\lambda_{g},T)\\ B=4\times 10^{-7}\cdot A\cdot K_{T}(\lambda_{b})Y(\lambda_{b})L(\lambda_{b},T)=K_{T}(\lambda_{b})K_{b}L(\lambda_{b},T) \end{array}\right.$$

where *λ_r_*, *λ_g_*, and *λ_b_* are the central wavelengths in the red, green, and blue channels, respectively. *L* (*λ, T*) is the flame brightness. *T* is the flame temperature. *A* is a transfer coefficient. *Y (λ*) is the response function. Since the response of the blue channel of the high-speed camera used in the experiments was so weak that the temperature measurement error was relatively large, the flame temperature measurement of the burning droplets was based on the calculation using the responses of the red and green channels. 

**Figure 12:**
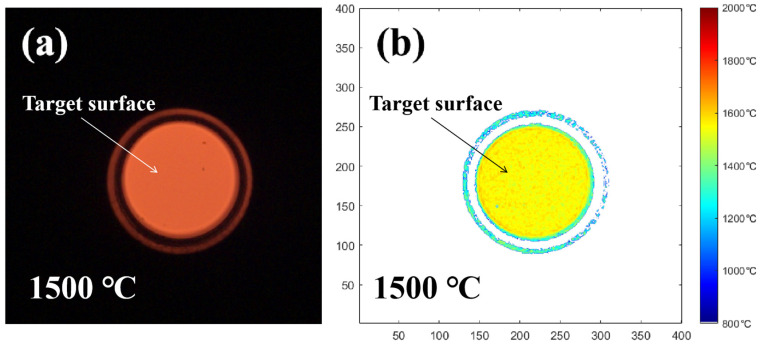
The target picture of the blackbody furnace at 1500 °C (**a**), and the corresponding measured temperature distribution (**b**).

According to Planck’s law, the simplified flame temperature *T* can be derived as:

(3) $$T=\frac{C_{2}\left(\frac{1}{\lambda_{g}}-\frac{1}{\lambda_{r}}\right)}{\ln\frac{R}{G}+5\ln\frac{\lambda_{r}}{\lambda_{g}}-\ln\frac{K_{r}}{K_{g}}-\ln\frac{K_{T}(\lambda_{r})}{K_{T}(\lambda_{g})}-\ln\frac{\varepsilon (\lambda_{r},T)}{\varepsilon (\lambda_{g},T)}}$$

where *C*_2_ is the second Planck constant, *ε* is the spectral emissivity. Based on the matrix operation and self-programming, the two-dimensional temperature distribution of the flame was obtained. On the basis of the measurement principle stated above, a calibration procedure had to first be executed by the blackbody furnace. As shown in Figure 12a, the target surface of the blackbody furnace was 1500 °C; the corresponding temperature distribution of the target surface captured by the high-speed camera is shown in Figure 12b. The highest relative error among all the pixels was less than 3.26%, which is an acceptable level.

**Figure 13:**
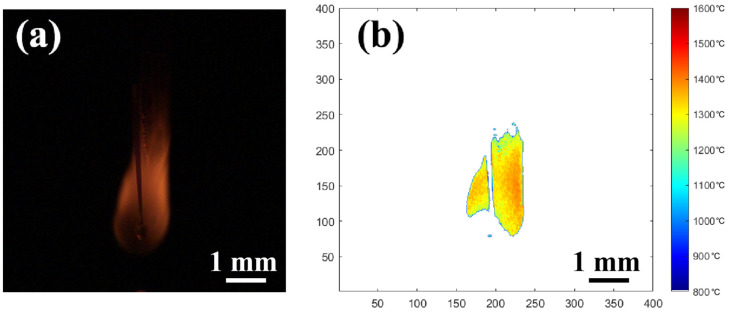
The flame picture of a burning droplet (**a**), and the corresponding calculated temperature distribution (**b**).

The flame temperature of the burning droplets was measured by the calibrated high-speed camera. Figure 13a shows a flame picture of a burning JP-10 droplet, and the calculated temperature distribution of the burning droplet is shown in Figure 3b.

It is worth noting that the droplet and flame structure was imaged by the high-speed camera under backlight illumination, while the flame temperature was measured by the radiation methodology based on the high-speed camera without backlight illumination. For each fuel formula listed in Table 1, 5~7 repeated experimental runs were conducted as the illumination was switched on or off, respectively. 

The squared diameter error of a burning droplet can be estimated as [33]:

(4) $$\frac{{∆d}^{2}}{d^{2}}=\frac{2}{3}\times \frac{∆v}{v}$$

in which *d* and *v* are the droplet diameter and volume, respectively. The initial droplet volume was 1 μL, and the actual volume of the droplet calculated by our digital image processing was *v* = 1.0531 mm^3^. Thus, the uncertainty of the droplet’s squared diameter can be calculated as:

(5) $$\frac{{∆d}^{2}}{d^{2}}=\frac{2}{3}\times \frac{∆v}{v}=\frac{2}{3}\times \frac{v-1}{v}=3.36\%$$

## 4. Conclusions

This work investigated the combustion characteristics of JP-10, JP-10/OA, JP-10/Al, and Al/JP-10/OA fuel droplets and conducted a comparison among them. The concentration effects of adding OA surfactant and Al nanoparticles on the combustion characteristics of JP-10 droplets were evaluated, especially for secondary atomization and micro-explosion. The conclusions can be made as follows:

1. The combustion of pure JP-10 fuel droplets followed the *d*^2^ law for the whole process, while the JP-10/OA, JP-10/Al, and Al/JP-10/OA fuel droplets followed the *d*^2^ law only in the stable stage. The addition of OA surfactant inhibited evaporation and reduced the effective burning rate of JP-10. Adding OA surfactant at high concentrations resulted in a large reduction in the combustion rate of droplets (8.7% @ 5.0 wt.% OA; 22.7% @ 12.5 wt.% OA). The addition of Al nanoparticles promoted evaporation and increased the effective burning rate of JP-10. The addition of both OA surfactant and Al nanoparticles had a synergetic effect on the evaporation and burning rate. At the optimum ratio of OA to Al for the best suspension stability, there was a turning point from promotion to inhibition with the increase in the Al concentration, and the critical value was 1.0 wt.%. When the Al concentration was greater than 1.0 wt.%, the combustion rate of the Al/JP-10/OA nanofluid fuel droplets decreased with the increase in the Al concentration but was lower than the combustion rate of the JP-10 droplets (8.5% reduction @ 5.0 wt.% OA + 2.5 wt.% Al; 40.8% reduction @ 12.5 wt.% OA + 5.0 wt.% Al).
2. The addition of OA surfactant could induce the occurrence of secondary atomization and micro-explosion of the droplets, but the critical concentration of OA needed to approximate or exceed 5.0 wt.%. The addition of Al nanoparticles at any concentration could result in the occurrence of secondary atomization and micro-explosion. The concentration of Al nanoparticles had a weak effect on the intensity of secondary atomization and micro-explosion. However, the addition of both of them greatly promoted secondary atomization and micro-explosion, even at dilute or dense concentrations.
3. Secondary atomization and micro-explosion could break up the mother droplets into a large number of finer daughter droplets and distribute them in the flame zone, which made the base fuel, the surfactant, and the nanoparticles burn more completely and improved the combustion performance. The unsteady combustion induced by secondary atomization and micro-explosion elevated the transient temperature by hundreds of Kelvins (compared to the stable flame temperature (~1400 °C), the highest temperature in the secondary atomization and micro-explosion zones increased by 12.85%~41.36%) and widened the high-temperature flame zone, which is beneficial for energy release.

Future work will focus on the study of the secondary atomization and micro-explosion characteristics of high-concentration Al/JP-10/OA nanofluid fuels (with an Al nanoparticle concentration of 30 wt.% and above).

## Figures and Tables

**Table 1:** The compositions of the prepared fuel samples.

No.	Sample’s Name	Compositions		
		JP-10 (wt.%)	OA (wt.%)	Al (wt.%)
1	JP-10	100.0	0.0	0.0
2	JP-10+OA0.2	99.8	0.2	0.0
3	JP-10+OA1.0	99.0	1.0	0.0
4	JP-10+OA2.0	98.0	2.0	0.0
5	JP-10+OA5.0	95.0	5.0	0.0
6	JP-10+OA12.5	87.5	12.5	0.0
7	JP-10+Al0.1	99.9	0.0	0.1
8	JP-10+Al0.5	99.5	0.0	0.5
9	JP-10+Al1.0	99.0	0.0	1.0
10	JP-10+Al2.5	97.5	0.0	2.5
11	JP-10+Al5.0	95.0	0.0	5.0
12	JP-10+OA+Al0.1	99.7	0.2	0.1
13	JP-10+OA+Al0.5	98.5	1.0	0.5
14	JP-10+OA+Al1.0	97.0	2.0	1.0
15	JP-10+OA+Al2.5	92.5	5.0	2.5
16	JP-10+OA+Al5.0	82.2	12.5	5.0

## Data Availability

Data could be available on queries.

## Supplementary Materials

The following supporting information can be downloaded at: https://www.mdpi.com/article/10.3390/molecules29081806/s1, Video S1: Video S1 in SI Pure JP-10; Video S2: Video S2 in SI JP-10+OA 0.2; Video S3: Video S3 in SI JP-10+OA 2.0; Video S4: Video S4 in SI JP-10+OA 12.5; Video S5: Video S5 in SI JP-10+Al 0.1; Video S6: Video S6 in SI JP-10+Al 1.0; Video S7: Video S7 in SI JP-10+Al 5.0; Video S8: Video S8 in SI JP-10+OA+Al 0.1; Video S9: Video S9 in SI JP-10+OA+Al 1.0; Video S10: Video S10 in SI JP-10+OA+Al 5.0.
